# Supplementary material for: Early swallowing rehabilitation and promotion of total oral intake in patients with aspiration pneumonia: A retrospective study
Source: PLoS One. 2024 Jan 19;19(1):e0296828. doi: 10.1371/journal.pone.0296828 (PMC10798484; doi:10.1371/journal.pone.0296828)
Supplement: Attachment — Submitted filename: Response to Reviewers.docx [file pone.0296828.s001.docx]

**Point-by-point response**

(Referees’ comments in bold; response in plain text; revised text in italic)

**Response to Editor**

We added some new references in the revised manuscript according to the reviewers’ suggestions as follows:

4. Marengoni A, Angleman S, Melis R, Mangialasche F, Karp A, Garmen A, et al. Aging with multimorbidity: a systematic review of the literature. Ageing Res Rev. 2011 Sep;10(4):430-9.

5. Vetrano DL, Palmer K, Marengoni A, Marzetti E, Lattanzio F, Roller-Wirnsberger R, et al. Joint Action ADVANTAGE WP4 Group. Frailty and Multimorbidity: A Systematic Review and Meta-analysis. J Gerontol A Biol Sci Med Sci. 2019 Apr 23;74(5):659-666.

6. Inouye SK, Westendorp RG, Saczynski JS. Delirium in elderly people. Lancet. 2014 Mar 8;383(9920):911-22. doi: 10.1016/S0140-6736(13)60688-1. Epub 2013 Aug 28.

7. Marcantonio ER. Delirium in Hospitalized Older Adults. N Engl J Med. 2017 Oct 12;377(15):1456-1466.

8. Ahn J, Chang JS, Kim JW. Postoperative Pneumonia and Aspiration Pneumonia Following Elderly Hip Fractures. J Nutr Health Aging. 2022;26(7):732-738.

9. Langmore SE, Skarupski KA, Park PS, Fries BE. Predictors of aspiration pneumonia in nursing home residents. Dysphagia. 2002 Fall;17(4):298-307.

12. Maniaci A, Lechien JR, La Mantia I, Iannella G, Ferlito S, Albanese G, et al. Cognitive Impairment and Mild to Moderate Dysphagia in Elderly Patients: A Retrospective Controlled Study. Ear Nose Throat J. 2022 Mar 7:1455613211054631.

13. Ueshima J, Shimizu A, Maeda K, Uno C, Shirai Y, Sonoi M, et al. Nutritional Management in Adult Patients With Dysphagia: Position Paper From Japanese Working Group on Integrated Nutrition for Dysphagic People. J Am Med Dir Assoc. 2022 Oct;23(10):1676-1682.

14. Okuni I, Ebihara S. Are Oropharyngeal Dysphagia Screening Tests Effective in Preventing Pneumonia? J Clin Med. 2022 Jan 13;11(2):370.

16. Jukic Peladic N, Orlandoni P, Di Rosa M, Giulioni G, Bartoloni L, Venturini C. Multidisciplinary Assessment and Individualized Nutritional Management of Dysphagia in Older Outpatients. Nutrients. 2023 Feb 22;15(5):1103

20. Charlson ME, Pompei P, Ales KL, MacKenzie CR. A new method of classifying prognostic comorbidity in longitudinal studies: development and validation. J Chronic Dis. 1987;40(5):373-83.

22. Szabó M, Kardos Z, Oláh C, Tamáska P, Hodosi K, Csánky E, et al. Severity and prognostic factors of SARS-CoV-2-induced pneumonia: The value of clinical and laboratory biomarkers and the A-DROP score. Front Med (Lausanne). 2022 Jul 22;9:920016.

We deleted one reference below in the revised manuscript.

Kadakia SC, Sullivan HO, Starnes E. Percutaneous endoscopic gastrostomy or jejunostomy and the incidence of aspiration in 79 patients. Am J Surg. 1992 Aug;164(2):114-8.

**Response to Reviewer: 1**

**Comment 1: When referring to neurodegenerative diseases and stroke as risk factor for aspiration pneumonia, the authors should also mention the impact of delirium in old hospitalized patients that may contribute to the risk to develop aspiration pneumonia (doi: 10.1007/s00134-021-06503-1 - doi: 10.3390/jcm12020435) as well as comorbidities (cardiovascular more frequently) that may lead to the fraility of old patients (doi: 10.1161/CIRCRESAHA.111.246876 - doi: 10.1111/echo.15462 - doi: 10.1038/s41569-018-0064-2). Please discuss and add these 5 references.**

Response 1: Thank you for reviewing our manuscript and giving us useful comment. We agree with the reviewer 1 that we should mention delirium and comorbidities; therefore, we revised the introduction as follows:

Page 3, lines 42-45

*Older adults have several risks for aspiration pneumonia. For example, common problems in older patients such as comorbidities [4], which can be associated with frailty [5], and delirium [6,7] were reported as the risk factors for aspiration pneumonia in hospitalized patients [8] or nursing home residents [9].*

**Comment 2: Did authors asked for a waiver by the local ethical committee to perform this study? Please specify.**

Response 2: We added sentences related to the waiver of written informed consent as follows:

Page 9, lines 160-163

*The ethics committee approved a waiver for written informed consent from each participant on the condition that we used an opt-out method to inform the study of the eligible participants. We disclosed the information about the study on the hospital’s website.*

**Comment 3: Please add the reduced number of included patients and the retrospective design as limitations of the study.**

Response 3: We added some sentences related to the reduced number of included patients and the retrospective design in the limitation section as follows:

Page 17, lines 269-274

*Third, because this was a retrospective design, some variables were missed. We used the multiple imputations by chained equations method to impute the missing values for the logistic regression models to reduce the effects of missing variables; however, these variables could still bias the study results. Fourth, the number of included patients was reduced due that strict criteria for exclusion and retrospective fashion of the study; therefore, some statistical analyses were underpowered.*

**Comment 4: Table 3 is hard to read. Please use square brackets for interquartile range and replace “exclusion 6” with “6 patients excluded”**

Response 4: We revised Table 3 according to the comment of the reviewer 1 (Page 13-14).

**Response to Reviewer: 2**

**Comment 1: Introduction. Preventing dysphagia in individuals with cognitive deterioration involves early identification of swallowing difficulties and a multidisciplinary approach to management. Interventions such as tailored swallowing exercises, dietary modifications, and proper oral care can mitigate the risk of dysphagia. Ongoing monitoring and regular reevaluation of swallowing function are crucial to ensure timely adjustments to the care plan and maintain the patient's quality of life., please discuss and cite doi:10.1177/01455613211054631.**

Response 1: Thank you for reviewing our manuscript and giving us useful comments. We agree with the reviewer that we should discuss prevention for dysphagia and its complications. We added the discussion with the reference that the reviewer recommended.

Page 3-4, lines 51-55

*Therefore, early identification of swallowing difficulties by ongoing monitoring and regular reevaluation of swallowing function [14] and multidisciplinary approach with registered dietitians, nutritionists, and speech therapists for tailored swallowing exercises, dietary modifications, and oral care that can mitigate the risk of dysphagia seem important for preventing aspiration pneumonia [16].*

**Comment 2: The introduction is quite lengthy and could be shortened to improve readability and retain the reader's attention.**

Response 2: According to the reviewer’s advice, we revised the introduction.

Page 3-4, lines 35-65

*Aging is associated with various health problems in the world. In some countries, the rate of aging (the rate of those aged 65 or over) is exceedingly high; it was reported to be 29.8%, the highest, in Japan in 2021 [1]. Meanwhile, pneumonia and aspiration pneumonia have become more common causes of death in the aging world [2], with reports showing that 76% of deaths from aspiration pneumonia occur in people aged 75 years and over [3]. Therefore, aspiration pneumonia in older adults is a major health concern.*

*Older adults have several risks for aspiration pneumonia. For example, common problems in older patients such as comorbidities [4], which can be associated with frailty [5], and delirium [6,7] were reported as the risk factors for aspiration pneumonia in hospitalized patients [8] or nursing home residents [9]. Furthermore, neurological diseases (e.g., stroke) and neurodegenerative diseases (e.g., Parkinson’s disease), cognitive dysfunction, cancer, sarcopenia, and aging itself [10,11,12,13], which are common in older population, can be associated with reduced swallowing function that is a key factor in the development of aspiration pneumonia [14]. Indeed, oropharyngeal dysphagia was reported to be present in approximately 90% of older patients diagnosed with pneumonia [15]. Therefore, early identification of swallowing difficulties by ongoing monitoring and regular reevaluation of swallowing function [14] and multidisciplinary approach with registered dietitians, nutritionists, and speech therapists for tailored swallowing exercises, dietary modifications, and oral care that can mitigate the risk of dysphagia seem important for preventing aspiration pneumonia [16].*

*As the treatment for aspiration pneumonia in acute settings, oral care and early rehabilitation are considered as important as antimicrobial and oxygen supplementation therapies. Indeed, previous studies suggested that early swallowing assessment and rehabilitation start was associated with less deterioration in swallowing function, lower in-hospital mortality rates, and higher total oral intake rates at discharge [17,18,19]. However, whether implementation of early swallowing assessment and rehabilitation can improve these outcomes in patients hospitalized due to aspiration pneumonia. Therefore, we conducted this study to assess the efficacy of a quality improvement action with early swallowing rehabilitation for patients with aspiration pneumonia in terms of total oral intake rates at discharge and in-hospital mortality.*

**Comment 3: To prevent dysphagia in cases of gastritis and malnutrition, it is essential to adopt a well-balanced diet tailored to the individual's specific needs and tolerances. Working with a registered dietitian can help create a customized nutrition plan that addresses gastritis symptoms and supports optimal nutrient intake. Additionally, incorporating swallowing therapy and exercises can further reduce the risk of dysphagia and promote safe and efficient eating habits., please discuss and cite doi:10.1002/lary.29890**

Response 3: We agree with the reviewer that multidisciplinary approach for reducing the risk of dysphagia. We addressed the issue in the introduction with adding a new reference.

Page 3-4, lines 51-55

*Therefore, early identification of swallowing difficulties by ongoing monitoring and regular reevaluation of swallowing function [14] and multidisciplinary approach with registered dietitians, nutritionists, and speech therapists for tailored swallowing exercises, dietary modifications, and oral care that can mitigate the risk of dysphagia seem important for preventing aspiration pneumonia [16].*

**Comment 4: Some statistical information (e.g., percentages) might be better presented as visual aids like tables or graphs for easier comprehension. Summarize the introduction by focusing on the main points and removing redundant information. Include visual aids, such as tables or graphs, to present statistical data more effectively.**

Response 4: According to the reviewer’s advice, we provided the tables 2-4 for presenting statistical information. We also summarized the introduction by focusing on the main points and removing redundant information.

Page 3-4, lines 35-65

*Aging is associated with various health problems in the world. In some countries, the rate of aging (the rate of those aged 65 or over) is exceedingly high; it was reported to be 29.8%, the highest, in Japan in 2021 [1]. Meanwhile, pneumonia and aspiration pneumonia have become more common causes of death in the aging world [2], with reports showing that 76% of deaths from aspiration pneumonia occur in people aged 75 years and over [3]. Therefore, aspiration pneumonia in older adults is a major health concern.*

*Older adults have several risks for aspiration pneumonia. For example, common problems in older patients such as comorbidities [4], which can be associated with frailty [5], and delirium [6,7] were reported as the risk factors for aspiration pneumonia in hospitalized patients [8] or nursing home residents [9]. Furthermore, neurological diseases (e.g., stroke) and neurodegenerative diseases (e.g., Parkinson’s disease), cognitive dysfunction, cancer, sarcopenia, and aging itself [10,11,12,13], which are common in older population, can be associated with reduced swallowing function that is a key factor in the development of aspiration pneumonia [14]. Indeed, oropharyngeal dysphagia was reported to be present in approximately 90% of older patients diagnosed with pneumonia [15]. Therefore, early identification of swallowing difficulties by ongoing monitoring and regular reevaluation of swallowing function [14] and multidisciplinary approach with registered dietitians, nutritionists, and speech therapists for tailored swallowing exercises, dietary modifications, and oral care that can mitigate the risk of dysphagia seem important for preventing aspiration pneumonia [16].*

*As the treatment for aspiration pneumonia in acute settings, oral care and early rehabilitation are considered as important as antimicrobial and oxygen supplementation therapies. Indeed, previous studies suggested that early swallowing assessment and rehabilitation start was associated with less deterioration in swallowing function, lower in-hospital mortality rates, and higher total oral intake rates at discharge [17,18,19]. However, whether implementation of early swallowing assessment and rehabilitation can improve these outcomes in patients hospitalized due to aspiration pneumonia. Therefore, we conducted this study to assess the efficacy of a quality improvement action with early swallowing rehabilitation for patients with aspiration pneumonia in terms of total oral intake rates at discharge and in-hospital mortality.*

**Comment 5: Methods. The methods section is somewhat dense and may be difficult for readers to follow. Some terminology and abbreviations are used without prior explanation (e.g., FILS, A-DROP scores), which could confuse readers.**

Response 5: We added explanations for special terminologies as follows:

Page 7-8, lines 126-135

*CCI is the Comorbidity Index that consists of 19 items corresponding to different medical comorbid conditions. The total score of the CCI consists in a simple sum of the weights, with higher scores indicating not only a greater mortality risk but also more severe comorbid conditions [20]. FILS is an ordinal scale to assess eating status, ranged 1 to 10: level 1-3, no oral intake; level 4-6, oral intake and alternative feeding; level 7-9 oral intake only; and level 10, normal [21]. A-DROP is a scoring system that expresses the severity of pneumonia, which includes Age (≥70 years in males and ≥ 75 years in females), Dehydration (BUN ≥ 7.5 mmol/l), Respiratory failure (SaO2 ≤ 90% or PaO2 ≤ 60 mmHg), Orientation disturbance (confusion) and low blood Pressure (systolic blood Pressure ≤ 90 mmHg) [22].*

**Comment 6: Divide the methods section into subsections such as "Study Design," "Participants," "Intervention," and "Data Collection" for improved readability.**

Response 6: According to the reviewer’s advice, we divided the methods section into “Study Design”, “Intervention and Participants” and “Data Collection”.

**Comment 7: Define and explain any terminology or abbreviations before using them in the text.**

Response 7: We spelled out all of the terminologies and abbreviations before using them and provided explanations for some terminologies.

Page 7-8, lines 119-135

*Charlson comorbidity index (CCI), whether the patient was at home before hospitalization, eating independence before admission (independent, partially, or fully assisted), Food Intake LEVEL Scale (FILS) before admission and at discharge, pneumonia severity (A-DROP scores), serum albumin level on admission and at discharge (g/dL), body mass index (BMI) on admission (kg/m2), time between admission and swallowing training initiation, time from admission to oral intake initiation, and in-hospital death.*

*CCI is the Comorbidity Index that consists of 19 items corresponding to different medical comorbid conditions. The total score of the CCI consists in a simple sum of the weights, with higher scores indicating not only a greater mortality risk but also more severe comorbid conditions [20]. FILS is an ordinal scale to assess eating status, ranged 1 to 10: level 1-3, no oral intake; level 4-6, oral intake and alternative feeding; level 7-9 oral intake only; and level 10, normal [21]. A-DROP is a scoring system that expresses the severity of pneumonia, which includes Age (≥70 years in males and ≥ 75 years in females), Dehydration (BUN ≥ 7.5 mmol/l), Respiratory failure (SaO2 ≤ 90% or PaO2 ≤ 60 mmHg), Orientation disturbance (confusion) and low blood Pressure (systolic blood Pressure ≤ 90 mmHg) [22].*

**Comment 8: Organize the content using subheadings: Divide the results into subsections with appropriate subheadings such as "Baseline Characteristics," "Swallowing Assessment and Oral Intake," and "Clinical Outcomes." This will make it easier for readers to follow the results and find the information they are interested in.**

Response 8: According to the reviewer’s advice, we divided results into some subsections such as “Baseline Characteristics”, “Swallowing Assessment and Oral Intake” and “Clinical Ourcomes”.

**Comment 9: Visual aids: Include tables or graphs to present the numerical data more effectively. Visual aids can help readers grasp the information quickly and enhance their understanding of the study's findings.**

Response 9: According to the reviewer’s advice, we provided tables to present the numerical data (Table 2-4).

**Comment 10: Clarify abbreviations and terminology: If not previously explained in the methods section, provide definitions for abbreviations and terms used in the results section (e.g., FILS, A-DROP scores). This will ensure that readers have a clear understanding of the concepts discussed.**

Response 10: According to the previous suggestion by the reviewer, we provided explanations for all abbreviations and terms in the Methods section.

**Comment 11: Emphasize significant findings: Highlight significant findings and differences between groups to help readers quickly identify the study's main outcomes. This can be done by using bold or italic formatting, or by summarizing significant results in a separate paragraph.**

Response 11: We apologize if we are out of line for saying this, but the guidelines of PlosOne seems not allowing the use of bold or italic for emphasizing the results. Therefore, though in all fairness, we summarized significant results in the first paragraph of the discussion section as follows:

Page 14-15, lines 213-218

*In this study, the implementation of a new protocol that aimed to facilitate early swallowing assessment and oral intake of patients admitted with aspiration pneumonia was significantly associated with a lower odds ratio for in-hospital mortality and no recovery to total oral intake than that before the implementation. In detail, the logistic regression analyses showed that the protocol intervention was significantly associated with a lower risk of composite outcomes (odds ratio, 0.22, 95%CI, 0.08-0.61, p=0.004).*

**Comment 12: Discuss limitations and future research: In the discussion section, address the limitations of the study and provide suggestions for future research. This helps to put the results into context and gives readers a better understanding of the study's implications.**

Response 12: According to the reviewer’s advice, we added some limitations and future perspectives as follows:

Page 17, lines 262-279

*This study had several limitations. First, this was a single-center study which may have limited the generalizability of the results. In particular, it was important that this study was conducted in a situation where a multidisciplinary team had already worked to initiate early rehabilitation for patients with aspiration pneumonia prior to the introduction of the protocol. Second, this study excluded patients who were not on a total oral intake prior to admission or patients who met the criteria for a high-risk profile to initiate oral intake; therefore, the effect of protocol implementation on such populations remains unknown. Third, because this was a retrospective design, some variables were missed. We used the multiple imputations by chained equations method to impute the missing values for the logistic regression models to reduce the effects of missing variables; however, these variables could still bias the study results. Fourth, the number of included patients was reduced due that strict criteria for exclusion and retrospective fashion of the study; therefore, some statistical analyses were underpowered. Future researches are warranted to assess whether an early swallowing rehabilitation intervention with the similar protocol can increase the rate of total oral intake at discharge and reduce the rate of in-hospital morality in other institutions, as well as whether this approach can be associated with the better these outcomes even when including patients with more higher-risk for dysphagia and its complications.*
